# Digital inclusive finance, green technological innovation, and carbon emissions from a spatial perspective

**DOI:** 10.1038/s41598-024-59081-9

**Published:** 2024-04-11

**Authors:** Yang Lu, Ziyi Xia

**Affiliations:** School of Economics, Jiangxi University of Finance and Economics, Nanchang, 330013 China

**Keywords:** Digital inclusive finance, Green technological innovation, Carbon emissions, Spatial Durbin model, Mediating effect, Climate-change impacts, Environmental economics, Sustainability

## Abstract

Based on the panel data of 276 prefecture-level cities in China from 2011 to 2020, this study explores the impact of digital inclusive finance (DIF) on carbon emissions and the intrinsic mechanism of green technological innovation from a spatial perspective by constructing a spatial econometric model, a mediating effect model, and a threshold model. The results show that DIF significantly inhibits carbon emissions, exhibiting a spatial spillover effect. The transmission mechanism from a spatial perspective shows that green technological innovation plays a partial mediating role between DIF and carbon emissions, with the mediating effect accounting for approximately 59.47%. The heterogeneity analysis suggests that the impact of DIF on the reduction of carbon emissions is more pronounced in large and medium-sized cities and eastern regions. Further discussion reveals that the carbon reduction effect of DIF is also influenced by green technological innovation and industrial structure upgrading, showing threshold effects with marginal decreases and gradual increases, respectively.

## Introduction

In recent years, global warming has emerged as a pressing worldwide issue with consequential impacts on human society, economies, and ecosystems due to continuous climate change. The major contributor to greenhouse gas emissions is carbon dioxide^1^. Therefore, China has firmly committed to achieving carbon peaking by 2030 and carbon neutrality by 2060^2^. To achieve this “dual-carbon” goal, a focus on the dynamic evolution of carbon emissions and the influencing factors is imperative. Financial development, known for its crucial impact on the evolution of carbon dioxide, provides a significant avenue for promoting social productivity^3^. Compared to traditional finance, digital inclusive finance (DIF) presents a more ubiquitous and inclusive approach, which not only enhances social economies but also aids in reducing environmental pollution. On the one hand, DIF can provide financial support to small and medium-sized firms (SMEs) for their clean technology development, equipment upgrades, and energy efficiency improvement initiatives. This, in turn, facilitates the sustainable development of enterprises and industries while mitigating carbon emissions. On the other hand, DIF plays a pivotal role in supporting the development of carbon markets. Through digital trading platforms and financial instruments, it stimulates the financing and implementation of carbon emission rights trading and carbon offsetting projects, alleviating the pressure associated with carbon emissions.

As a cutting-edge financial approach that combines big data and information technologies, DIF not only holds the potential to impact carbon emissions directly but also can optimize various aspects of energy management and supply chain by supporting the innovation of green technology. This optimization aims to improve energy management, enhance resource utilization efficiency, and decrease carbon emissions. Furthermore, considering the spatial correlation of carbon emissions, neighboring cities can mutually influence each other in spatial terms^4^. Therefore, this study primarily investigates the impact of DIF on carbon emissions from a spatial perspective. It emphasizes that DIF affects carbon emissions not only directly but also indirectly through green technological innovation, which is significant for the growth of a sustainable green economy and the reduction of carbon emissions in China.

Valuable conclusions have been derived throughout the existing literature. Firstly, it has been found that DIF plays a favorable role in reducing carbon emissions^5,6^. Secondly, DIF contributes to the advancement of green technological innovation^7,8^. Lastly, green technological innovation is an effective means to curb carbon emissions^9,10^. However, previous studies have potential areas for improvement. Existing studies on the impact of DIF on carbon emissions have predominantly focused on the bidirectional relationship between DIF, green technological innovation, and carbon emissions and have not adequately explored the underlying mechanisms and their spatial correlation. This provides an opportunity for this study to make a modest but noteworthy contribution in this area. (1) This study thoroughly considers the spatial correlation of variables by empirically investigating the direct impact of DIF on carbon emissions and further testing the mediating mechanisms of green technological innovation based on the spatial Durbin model (SDM), enabling the conclusions to be more comprehensive and reliable. (2) From a spatial perspective, the heterogeneous influence of DIF on carbon emissions is explored based on the urban location and scale, enriching the theory and practice of DIF for sustainable green development. (3) By constructing a threshold effect model from a spatial perspective, this study further explores the nonlinear impacts of DIF on carbon emissions, which provides valuable references for policymakers to formulate scientific carbon emission policies.

Therefore, this study utilizes panel data from 276 Chinese cities from 2011 to 2020 and empirically examines the impact of DIF on carbon emissions and the underlying mechanism of green technological innovation, employing spatial econometric, threshold, and mediation models.

The rest of the research is organized as follows. The literature review and research hypotheses are presented in "Literature review and research hypotheses" section. The research model setting and variable definitions are introduced in "Research design" section. The empirical analysis results are described in "Empirical analysis" section. Conclusions and policy recommendations are provided in "Conclusions and policy recommendations" section.

## Literature review and research hypotheses

### Direct and spillover effects of DIF

In the process of financial development in China, DIF is broadly acknowledged as an effective strategy to foster sustainable economic growth, reduce carbon emissions, and address environmental externalities^11^. Specifically, DIF contributes to decreasing energy consumption and carbon emissions, manifested in two main aspects. (1) Regarding the inclusiveness of DIF, it provides diversified financial services to vulnerable groups in remote rural areas and underdeveloped regions by expanding the coverage of financial services^12^. This helps their access to clean technologies and renewable energy sources, thereby fostering the development of green consumption and production practices. Furthermore, by employing digital technology and innovative financial service models, DIF effectively lowers the cost and barriers of financial services^13^. This facilitates green and low-carbon technology enterprises in obtaining necessary financing and loan support, thus driving the growth of the low-carbon industry. (2) Considering the sustainability of DIF, it optimizes business processes and resource utilization efficiency through digital financial services and online transaction platforms^14^. This not only diminishes the reliance on paper documents in traditional finance, thereby reducing carbon emissions associated with paper consumption, but also provides portable financial services to consumers, thereby reducing carbon emissions associated with transportation. As a result, the overall energy consumption of financial activities is subsequently decreased.

In addition, the spatial spillover impact of DIF on carbon emissions primarily includes the following aspects: First, from a regional standpoint, DIF can transcend spatial and temporal constraints, facilitating “zero-distance” interaction between providers and consumers of financial products. Therefore, the evolution of DIF is subject to spatial externalities emanating from adjacent areas, indicating the presence of spatial spillover effects^15^. Second, in terms of the level of development in DIF, its growth in neighboring regions may lead to technology or capital spillover and generate radiation effects, thereby promoting local low-carbon economic growth^16^. Consequently, hypothesis 1 is proposed:

#### Hypothesis 1

The DIF can directly curb carbon emissions and have spatial spillover effects.

### The mediating mechanism of green technological innovation

DIF promotes green technological innovation, which, in turn, can reduce carbon emissions significantly^17,18^. Fundamentally, green technological innovation serves as a practical pathway to achieve carbon reduction. On the one hand, DIF utilizes scientific technologies such as data intelligence to form precise user profiles, analyze consumption habits, and monitor energy usage. This enables the provision of personalized energy management recommendations, suggestions for green products, and carbon footprint tracking services. Moreover, DIF can proactively prioritize environmental indicators, including green and low-carbon criteria, to serve as the basis for loan evaluation, guiding users to reduce their carbon emissions actively. On the other hand, DIF can provide financial support for SMEs in implementing energy-saving and emission-reduction measures through leveraging energy-efficient technologies^19^. By offering financial assistance, such as low-interest loans and technical advice, SMEs are better equipped to adopt energy-efficient equipment and renewable energy technologies, reducing their reliance on traditional high-carbon energy sources and effectively lowering carbon emissions.

In addition, the spatial spillover impact of DIF on carbon emissions through green technology innovation usually involves the following approaches: First, green technological innovation depends on scientific research investment and financial strength, while different regions have different geographical locations and cultural development, the financial productivity is influenced by these factors to a certain extent and has a significant correlation with the spatial structure of cities^20^. Second, green technological innovation can produce demonstration effects^21^. The enhancement of green technological innovation capacity in adjacent regions exerts a specific demonstration effect on the region, and green technology within the region can strengthen its innovation capacity through the assimilation of advanced technology and the accumulation of innovation experience, which will facilitate the diffusion of low-carbon technology. Therefore, hypothesis 2 is proposed:

#### Hypothesis 2

The DIF can promote the reduction of carbon emissions through green technological innovation and exhibit spatial spillover effects.

### The nonlinear impact of DIF on carbon emissions

The growth of DIF provides vital funding for SMEs’ research and innovation efforts^22^. This support leads to reduced financing costs, enabling SMEs to enhance energy-saving technologies and energy management systems, thereby facilitating effective energy conservation and emission reduction. Moreover, DIF possesses attributes such as cross-temporality and high liquidity^23^, which, by breaking information barriers and market monopolies, facilitate easier collaboration in innovation activities between regions. This enhances the innovation output capacity of neighboring areas, consequently elevating the level of green technological innovation. Upon reaching a certain level, green technological innovation contributes to optimizing energy utilization, promoting the adoption of clean energy technologies, reducing dependence on fossil fuels, and ultimately decreasing carbon emissions. Furthermore, advancements in green technological innovation encourage people to adopt environmentally friendly and sustainable consumption patterns, pressuring manufacturers and high-pollution enterprises to increase their market demand for green and low-carbon technologies^24^, resulting in reduced carbon emissions.

**Figure 1:**
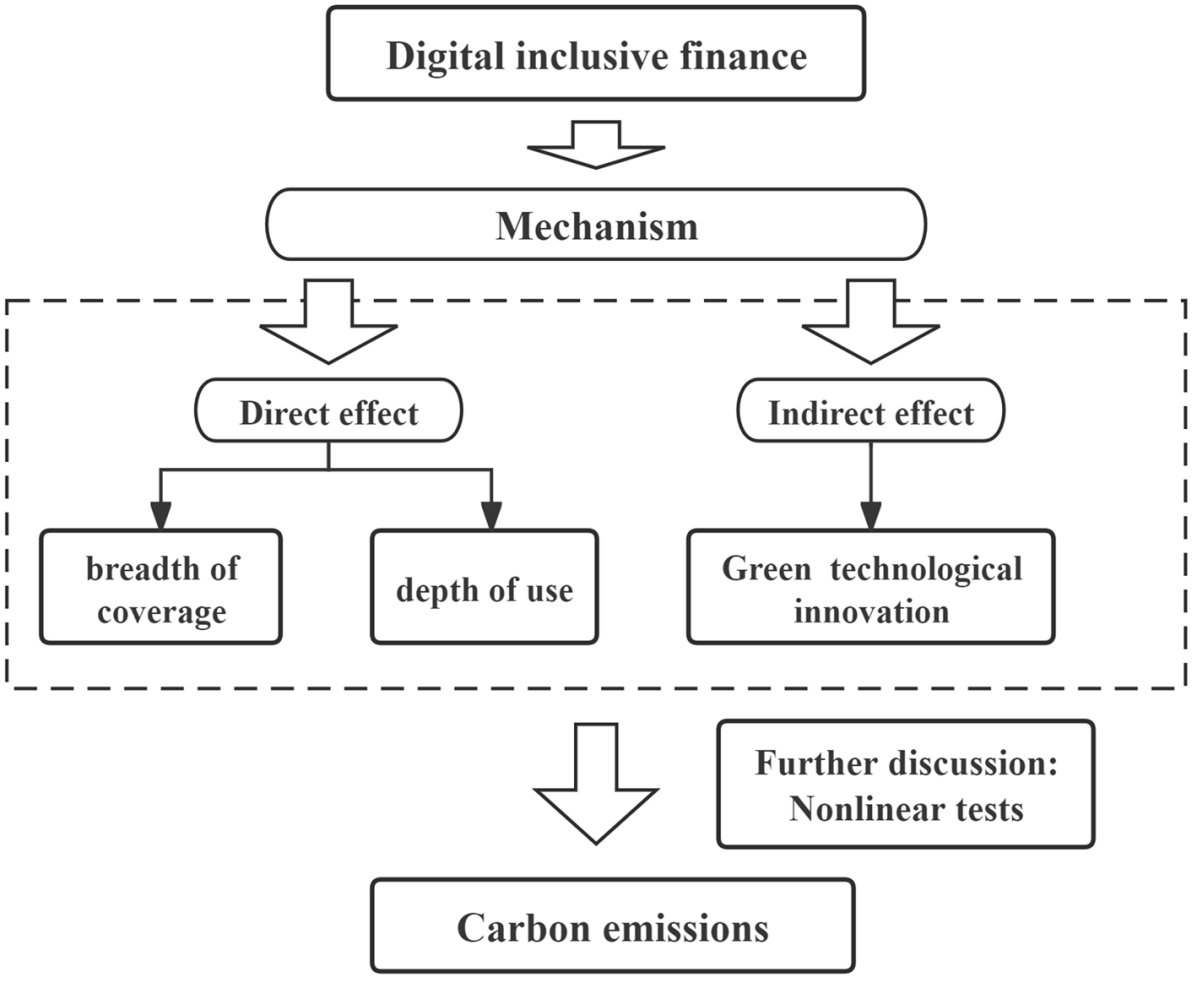
The impact mechanism of DIF on carbon emissions.

In addition, DIF acts as a significant catalyst for industrial structure upgrading^25^. It enhances the efficiency of financial services and effectively mitigates the misallocation of financial resources, thus contributing to enhanced energy and production efficiency within industries and consequently reducing industrial pollution emissions^26^. Firstly, the initial upgrading of the industrial structure optimizes the energy consumption structure by promoting the substitution of renewable energy for traditional high-carbon energy sources, thereby reducing carbon emissions. Secondly, in advanced stages of development, this process enhances the allocation efficiency of production factors. It directs the flow of production factors towards more efficient and environmentally friendly industries, thus further promoting carbon mitigation. Accordingly, this study proposes hypothesis 3. Furthermore, the impact mechanism of DIF on carbon emissions is shown in Fig. 1.

#### Hypothesis 3

The impact of DIF on carbon emissions exhibits nonlinear effects under varying levels of green technological innovation and industrial structure upgrading.

## Research design

### Empirical model setting

#### Setting of the benchmark model

To examine the direct impact of DIF on carbon emissions, the following benchmark regression model is established:

1 $$ CI_{it} = \alpha_{0} + \alpha_{1} DIF_{it} + \beta X_{it} + \mu_{r} + v_{t} + \varepsilon_{it} $$

where $i$ and $t$ refer to the city and year, respectively; $CI_{it}$ represents the carbon emission; $DIF$ represents the DIF index; $\alpha_{0}$ represents the intercept term; $\alpha_{1}$ represents the coefficient of the impact of DIF on carbon emissions; $X_{it}$ represents the control variables; $\mu_{i}$ and $v_{t}$ indicate individual and time-fixed effects, respectively; and $\varepsilon_{it}$ represents the random disturbance term.

#### Setting of the spatial econometric model

Considering the spatial correlation and dependence among variables, this study employed the likelihood ratio (LR) and Hausman^27^ tests, which indicated that the SDM is more significant than other spatial econometric models. Therefore, the SDM is selected in this study and specified as follows:

2 $$ CI_{it} = \lambda_{0} + \rho_{1} \sum\limits_{i = 1}^{n} {w_{ij} CI_{jt} } + \lambda_{1} DIF_{it} + \rho_{2} \sum\limits_{i = 1}^{n} {w_{ij} DIF_{jt} } + \lambda_{2} X_{it} + \sum\limits_{i = 1}^{n} {\rho_{3} w_{ij} X_{jt} } + \mu_{i} + v_{t} + \varepsilon_{it} $$

In Eq. (2), $w_{ij}$ represents the spatial weight matrix, illustrating the spatial connections between various cities. The parameters $i$ and $j$ denote the area, and $t$ represents the year.

#### Setting of the mediating effect test

Drawing on Wen et al.^28^ and Baron and Kenny^29^, the stepwise regression method is utilized for empirical analysis. Based on the SDM, the mediating effect mechanism is thoroughly examined and analyzed from a spatial perspective. The specified model is presented below:

3 $$ \ln inn_{it} = \beta_{0} + \gamma_{1} \sum\limits_{n = 1}^{n} {w_{ij} } \ln inn_{it} + \beta_{1} DIF_{jt} \beta_{2} X_{it} + \gamma_{2} \sum\limits_{n = 1}^{n} {w_{ij} } DIF_{jt} + \beta_{2} X_{{_{it} }} + \gamma_{3} \sum\limits_{n = 1}^{n} {w_{ij} } X_{ij} + \mu_{i} + v_{t} + \vartheta_{it} $$

4 $$\begin{aligned} CI_{it} & = \delta_{0} + \theta_{1} \sum\limits_{i = 1}^{n} {w_{ij} } CI + \delta_{1} DIF_{it} + \theta_{2} \sum\limits_{i = 1}^{n} {w_{ij} } DIF_{jt} + \delta_{2} \ln inn_{it} + \theta_{3} \sum\limits_{i = 1}^{n} {w_{ij} } \ln inn_{it} \\ & \quad + \delta_{3} X_{ij} + \theta_{4} \sum\limits_{i = 1}^{n} {w_{ij} } X_{ij} + \mu_{i} + v_{t} + \tau_{it} \end{aligned}$$

Among them, $lninn$ represents green technological innovation; $\lambda_{0}$, $\beta_{0}$, and $\delta_{0}$ denote the estimated parameters; and the other variables stay unchanged as before. Equations (2) and (3) represent the impact of DIF on carbon emissions and green technological innovation, respectively. The mediating effect is examined in Eq. (4), considering green technological innovation as a mediating variable to analyze its influence mechanism.

### Setting of the indicators

#### Explained variable: carbon emissions (CI)

Referring to the method of Wu and Guo^30^ and utilizing available city-level data. This study considers various sources of carbon emissions, including carbon emissions generated by the consumption of electric energy, gas, natural gas, transportation, and thermal energy. Based on the corresponding carbon emission coefficients, the amount of carbon emissions that are required in the production of each energy source is calculated. The totals for each sub-category are aggregated to ascertain the overall carbon emissions of each prefectural-level city.

#### Explanatory variable: digital inclusive finance (DIF)

The DIF index from Peking University is utilized as an indicator, drawing on Guo et al.^31^. The sub-dimensional indices, namely the breadth of coverage (breadth), depth of use (depth), and the degree of digitization (digitization), are employed to further investigate the impact of DIF on urban carbon emissions. Furthermore, the DIF and its sub-dimensional indices have been logarithmically treated to mitigate heteroskedasticity.

#### Mediating variable: green technological innovation (lninn)

Drawing on Liu et al.^32^, green technological innovation is utilized as a mediating variable to measure its level using green patent application data, including invention and utility model patents. We primarily consider the following two aspects: First, the capacity for green innovation production is closely associated with the volume of green patent applications. A greater number of green patent applications signifies more frequent research and development (R&D) activities, thus having significant economic value as knowledge assets^33^. Second, green patent applications are closely related to R&D investment. Considering the lengthy nature of the green patent application process, a higher volume of green patent applications reflects more intense R&D investment. Therefore, using the volume of green patent applications as a metric offers a more effective and intuitive measure of green technological innovation.

#### Control variable

In light of the objective of this study and considering the various variables affecting carbon emissions, we have selected the following control variables, drawing on Wang and Guo^34^ and Lee and Wang^35^. (1) Economic development level (lngdp), measured by the logarithm of Gross Domestic Product (GDP). (2) Industrial structure upgrading (Stru), quantified by the proportion of the value-added of the secondary industry to GDP. (3) Financial development (Finance), measured by the ratio of deposit and loan balances of financial institutions to GDP. (4) Population density (Density), measured by the ratio of the registered population of cities to the administrative area. (5) Government support for technology (Tech), measured by the ratio of fiscal expenditure on science and technology to total fiscal expenditure in cities. (6) Foreign direct investment (lnfdi), measured as the logarithm of actual foreign investment.

### Data sources and descriptive statistics

The primary data for this study were sourced from the CNRDS database, the China City Statistical Yearbook, the China Statistical Yearbook, and the Digital Finance Research Center of Peking University. Additionally, to minimize the potential influence of outliers on empirical findings, this study tailors the sample data according to the top and bottom 1% (Winsor). The descriptive statistical characteristics of each variable are shown in Table 1.

**Table 1:** Descriptive statistics of the sample data.

Type	Variables	Obs	Mean	Std	Min	Max
Explained variable	CI	2760	0.109	0.142	0.002	1.166
Mediating variables	DIF	2760	5.057	0.511	3.173	5.750
	Breadth	2760	4.989	0.559	1.629	5.753
	Depth	2760	5.041	0.510	2.639	5.773
	Digitization	2760	5.218	0.607	1.817	5.823
Intermediary variable	lninn	2760	1.599	0.342	0.094	2.270
Control variables	lngdp	2760	16.619	0.892	14.497	19.337
	Stru	2760	1.213	0.552	0.208	3.913
	Finance	2760	2.462	1.138	0.732	7.354
	lnfdi	2760	11.843	1.950	4.929	16.121
	Tech	2760	0.017	0.016	0.001	0.118
	Density	2760	5.757	0.888	2.863	8.021
Robust variables	IP	2760	21.899	12.389	2.662	121.875
	Edu	2760	0.020	0.025	0.000	0.128
	Urban	2760	0.550	0.144	0.226	0.953

## Empirical analysis

### Benchmark regression

#### Benchmark regression analysis

Table 2 presents the results of the stepwise regression analysis of DIF on the sample. To explore the impact of DIF on carbon emissions more accurately, control variables were added sequentially in columns (2) to (7). Notably, the coefficient of DIF is significantly negative at the 1% level, suggesting that DIF has a significant inhibitory effect on carbon emissions. Among the control variables, upgrades in industrial structure, levels of foreign investment, and population density exhibit positive significance, implying that foreign investment and upgrades in industrial structure have propelled the expansion of production scales, leading to increased energy consumption and carbon emissions. The growing population density might also lead to increased greenhouse gas emissions. However, the coefficients of financial and economic development are negative but insignificant, probably because of the irrational energy consumption structure during its development, thereby failing to reduce carbon emissions effectively.

**Table 2:** Benchmark regression results.

	(1)	(2)	(3)	(4)	(5)	(6)	(7)
DIF	− 0.109***	− 0.109***	− 0.109***	− 0.092***	− 0.094***	− 0.088***	− 0.079***
	(− 5.14)	(− 5.13)	(− 5.12)	(− 4.71)	(− 4.79)	(− 4.62)	(− 4.53)
Finance		0.002	0.002	− 0.001	− 0.002	− 0.0019	− 0.002
		(0.23)	(0.19)	(− 0.09)	(− 0.12)	(− 0.15)	(− 0.13)
lngdp			0.001	− 0.026	− 0.037	− 0.045	− 0.047
			(0.03)	(− 0.92)	(− 1.28)	(− 1.46)	(− 1.52)
Stru				0.032***	0.034***	0.035***	0.030***
				(3.51)	(3.67)	(3.69)	(3.40)
lnfdi					0.004**	0.004**	0.004**
					(2.89)	(2.55)	(2.56)
Tech						0.530	0.426
						(1.43)	(1.17)
Density							0.067**
							(2.57)
Constant	0.495***	0.491***	0.481	0.807*	0.936**	1.046**	0.663
	(6.07)	(5.86)	(1.19)	(1.75)	(2.02)	(2.12)	(1.37)
Time fixed	Yes	Yes	Yes	Yes	Yes	Yes	Yes
Individual fixed	Yes	Yes	Yes	Yes	Yes	Yes	Yes
Observation	2760	2760	2760	2760	2760	2760	2760
R^2^	0.389	0.389	0.389	0.400	0.402	0.405	0.412

*p < 10%, **p < 5% and ***p < 1%, the same below.

#### Sub-dimensional test analysis

DIF is divided into three dimensions to further explore the influence of DIF on carbon emissions, as presented in Table 3. The findings indicate that both the breadth and depth of DIF can significantly decrease carbon emissions, whereas digitalization of DIF does not substantially inhibit carbon emissions. Specifically, column (1) in Table 3 indicates that the breadth of DIF significantly inhibits the level of urban carbon emissions at the 1% level, implying that as digital financial services spread farther and deeper, efficient allocation of resources and improved production efficiency become possible, thus reducing carbon emissions. Column (2) examines the impact of the depth of DIF on carbon emissions. The results indicate that the depth of DIF significantly reduces carbon emissions at the 5% level. Depth of use represents a vertical extension of the frequency and intensity of people’s engagement with DIF products, reflecting that the advantages of the low cost and portability of DIF can help support investment in green and low-carbon projects. Finally, column (3) suggests that digitalization does not contribute to reductions in carbon emissions. This may be attributed to the insufficient investment in network infrastructure, including digital computing, resulting in minimal impact on carbon emission reductions. However, in general terms, the overall development of DIF will undeniably still suppress urban carbon emissions.

**Table 3:** Decomposition regression results.

	(1)	(2)	(3)
Breadth	− 0.039***		
	(− 4.65)		
Depth		− 0.027**	
		(− 2.25)	
Digitization			0.014**
			(2.07)
Constant	0.475	0.576	0.402
Controls	Yes	Yes	yes
Time fixed effect	Yes	Yes	yes
Individual fixed effect	Yes	yes	yes
Observation	2760	2760	2760
R^2^	0.412	0.407	0.407

### Spatial effects analysis

#### Spatial correlation analysis

In line with the principles of spatial economics, it is essential to check the spatial correlation of each variable before constructing a spatial econometric model^36^. Therefore, the Moran index model is employed to examine the spatial correlation of each variable, which is set as follows:

5 $$ Moran^{\prime}s\,I = \frac{{\sum\nolimits_{i = 1}^{n} {\sum\nolimits_{j = 1}^{n} {w_{ij} \left( {Y_{I} - \overline{Y}} \right)} \left( {Y_{j} - \overline{Y}} \right)} }}{{\sum\nolimits_{i = 1}^{n} {\sum\nolimits_{j = 1}^{{}} {w_{ij} \left( {Y_{I} - \overline{Y}} \right)\left( {Y_{j} - \overline{Y}} \right)} } }} $$

where $Y_{i}$ and $Y_{j}$ represent the observed values in cities $i$ and $j$, respectively; $\overline{Y}$ represents the sample average value; $w_{ij}$ is the spatial weight matrix; $n$ represents the number of regions; and $I$ represents the global Moran’s I index. The global Moran index fits a normal distribution, suggesting that the larger the absolute value of the Moran I index, the stronger its correlation.

In this study, the adjacency weight matrix (W_1_) is set based on whether the cities are adjacent, and the inverse distance squared weight matrix (W_2_) is derived from the square of the inverse geographical distance between cities.

The findings in Table 4 show that Moran’s I indices for both core and explanatory variables are significantly positive under the spatial weight matrix, achieving statistical significance at the 1% level across all years. It indicates a significant positive spatial autocorrelation for both DIF and carbon emissions. Further analysis indicates that despite fluctuations, Moran’s I index for carbon emissions exhibits a relatively narrow range of variation, suggesting greater stability. Moreover, Moran’s I value for DIF shows an increasing trend, indicating a strengthening spatial correlation over time.

**Table 4:** Global Moran’s I values.

			2011	2012	2013	2014	2015	2016	2017	2018	2019	2020
DIF	W_1_	Moran’s I	0.077	0.095	0.111	0.078	0.089	0.094	0.109	0.140	0.150	0.162
		p-value	0.000	0.000	0.000	0.000	0.000	0.000	0.000	0.000	0.000	0.000
	W_2_	Moran’s I	0.354	0.357	0.332	0.325	0.329	0.316	0.369	0.425	0.439	0.459
		p-value	0.000	0.000	0.000	0.001	0.001	0.001	0.000	0.000	0.000	0.000
CI	W_1_	Moran’s I	0.021	0.021	0.023	0.021	0.022	0.021	0.039	0.039	0.041	0.039
		p-value	0.000	0.000	0.000	0.000	0.000	0.000	0.000	0.000	0.000	0.000
	W_2_	Moran’s I	0.219	0.252	0.251	0.277	0.298	0.288	0.317	0.292	0.277	0.231
		p-value	0.012	0.005	0.005	0.002	0.001	0.002	0.001	0.002	0.003	0.009

**Figure 2:**
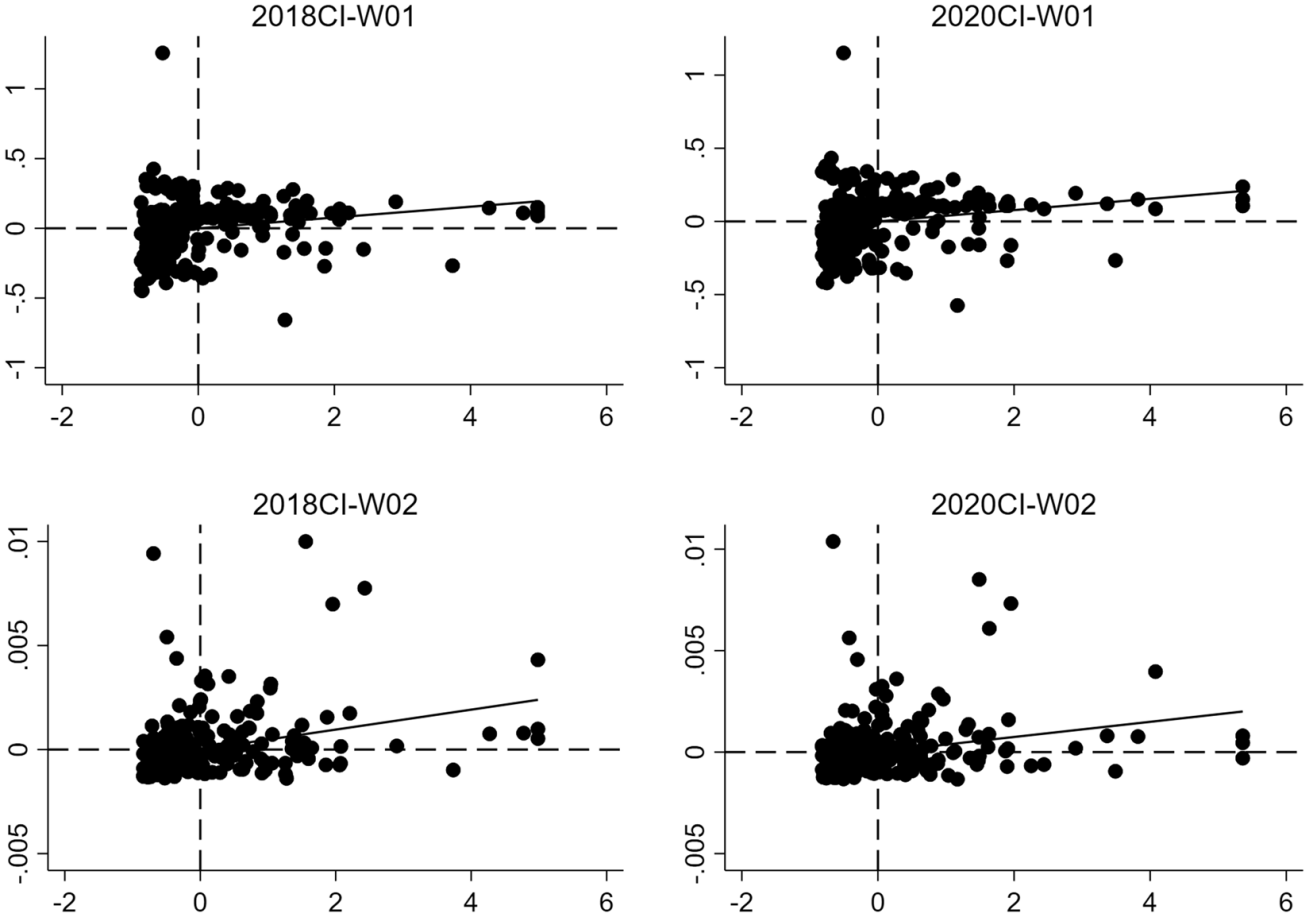
Local Moran scatter plot of carbon emissions in 2018 and 2020.

This study employs the LISA statistic to generate local Moran scatter plots for a more intuitive observation of spatial agglomeration among cities^37^. Figure 2 shows the Moran scatter plots of carbon emissions in 2018 and 2020. It can be seen that most of the carbon emission levels of each city in China are scattered in the first and third quadrants, indicating significant spatial agglomeration of carbon emissions within these cities, characterized by high-high and low-low agglomeration patterns.

Consequently, thoroughly considering spatial factors and employing spatial econometric models is critical in analyzing the impact mechanism of DIF on carbon emissions for a sound analysis.

#### Spatial econometric regression analysis

First, referring to the test of Elhorsta^38^, the LR test is used in this study to select the suitable estimation form for the spatial panel model, as presented in Table 5. The test results based on the spatial weight matrices W_1_ and W_2_ demonstrate that all statistics are significant at the 1% level, indicating that both LR tests have been passed and the null hypothesis is robustly rejected. Thus, the SDM is more appropriate than the spatial error model (SEM) and the spatial autoregressive model (SAR). Second, this study selects a double fixed effects model following a Hausman test of the spatial econometric model, which shows that the original hypothesis of random effects is firmly rejected at the 1% level for p-values under W_1_ and W_2_. Therefore, the SDM under the double fixed effects should be selected for analysis in this study.

**Table 5:** Regression results of the SDM.

Variables	(1) W_1_		(2) W_2_	
	Coefficient	t-statistic	Coefficient	t-statistic
DIF	− 0.040**	− 2.23	− 0.086***	− 4.98
W × DIF	− 0.402***	3.45	− 3.272*	− 1.68
Finance	0.029***	14.62	0.033***	16.79
lngdp	0.110***	34.07	0.112***	34.30
Stru	0.002	0.43	0.001	0.27
lnfdi	− 0.003*	− 1.76	− 0.001	− 0.61
Tech	1.097***	7.53	0.971***	5.87
Density	− 0.003	− 1.25	− 0.017***	− 7.06
Spatial-rho	0.208	1.63	34.994***	6.71
sigma2_e	0.008***	37.12	0.007***	37.05
Individual fixed effect	Yes		Yes	
Time fixed effect	Yes		Yes	
Observation	2760		2760	

The results of the spatial econometric models, Model (1) and Model (2), are presented in Table 6. The analysis reveals the following: First, under W_1_ and W_2_, the coefficients of the DIF and the interaction term W × DIF are significantly negative at the 1% level. This indicates that DIF markedly influences the reduction of carbon emissions locally and in neighboring areas. Second, the population density coefficient under W_2_ is significantly negative, indicating that a larger population may encourage a greater supply of technological innovation talent in the city, which in turn may reduce carbon emissions in the local area. Third, the coefficient of the control variable lngdp is significantly positive. This may be attributed to the current economic development pattern in China, which still relies on extensive industrial growth and exhibits limited effectiveness in curbing carbon emissions due to substantial energy consumption.

**Table 6:** LR and Hausman test results.

Type of weight matrix	W_1_		W_2_	
Index	X^2^	p-value	X^2^	p-value
SDM VS SAR	38.610	0.000	37.930	0.000
SDM VS SEM	37.290	0.000	35.680	0.000
Hausman test	142.710	0.000	421.180	0.000

#### Decomposition of spatial effects

The spatial effects are decomposed in this study to further analyze the direct, indirect, and total effects, as shown in Table 7. (1) The regression coefficient of the direct effect of DIF is significantly negative, suggesting that DIF has a crucial role in mitigating local carbon emissions. By promoting digital financial services, DIF makes it easier for people to access renewable energy and low-carbon products and reduces their dependence on traditional high-carbon energy sources, thus reducing carbon emissions. (2) The indirect effects of DIF are significant at the 1% and 5% levels, respectively, indicating significant spatial spillover effects on carbon emission reduction. It not only lowers local carbon emissions but also, due to geographical interconnections, drives the suppression of carbon emissions in neighboring regions. This can be attributed to the development of DIF overcoming geographic limitations, enabling the cross-regional transfer of low-carbon technologies via digital technology and others, thereby producing spatial spillover effects to some extent. (3) Under W_1_ and W_2_, the total effects of DIF are both statistically negative at the 1% level, with the indirect effect larger than the direct effect under W_1_ and the direct effect greater than the indirect effect under W_2_. It indicates that the spatial effects of DIF in mitigating carbon emissions are significantly influenced by adjacent spatial distances. This can be attributed to the broader coverage and greater accessibility of DIF in neighboring cities, where shorter distances enable swift dissemination and popularization of digital financial services. Furthermore, adjacent regions often share similar energy infrastructure and green energy networks, contributing to the efficacy of DIF and carbon emission reduction. Thus, hypothesis 1 is validated.

**Table 7:** Results of spatial spillover effects.

Spatial weight matrix		Direct effect	Indirect effect	Total effect
W_1_	DIF	− 0.040**	− 0.523***	− 0.563***
W_2_	DIF	− 0.087***	− 0.011**	− 0.098***

#### Analysis of the spatial mediation effects

From the standpoint of spatial effects decomposition, this study investigates the mechanism behind the mediating effect of green technological innovation. As shown in Table 8, columns (1), (2), and (3) correspond to the regression outcomes of Eqs. (2)–(4). The testing steps and outcomes for the mediating effects are presented below.

**Table 8:** Regression results of the mediating effects.

Variables	(1)			(2)			(3)		
	Direct	Indirect	Total	Direct	Indirect	Total	Direct	Indirect	Total
DIF	− 0.039**	− 0.328**	− 0.368***	0.414***	1.138***	1.552***	− 0.090***	− 0.025*	− 0.068**
	(− 2.15)	(− 2.38)	(− 2.76)	(13.01)	(6.66)	(9.53)	(− 8.05)	(− 1.95)	(− 2.39)
lninn							− 0.043**	− 0.051***	− 0.141***
							(− 2.51)	(− 3.28)	(− 6.17)
Finance	0.028***	0.096***	0.125***	0.023***	0.094***	0.117***	0.037***	0.021***	0.058***
	(14.65)	(5.71)	(7.47)	(6.90)	(5.22)	(6.71)	(20.54)	(3.64)	(9.15)
lngdp	0.110***	0.022	0.132***	0.204***	− 0.065*	− 0.139***	0.131***	0.075***	0.207***
	(35.36)	(0.64)	(3.81)	(38.15)	(− 1.65)	(− 3.55)	(33.36)	(3.64)	(9.54)
Stru	0.001	0.198***	0.199***	0.007	0.346***	− 0.353***	0.003	0.002	0.005
	(0.27)	(4.76)	(4.78)	(1.03)	(7.85)	(− 8.08)	(0.82)	(0.75)	(0.80)
lnfdi	− 0.002*	0.059***	0.057***	0.004	0.021*	0.024**	− 0.002*	− 0.001	− 0.004
	(− 1.62)	(5.32)	(5.17)	(1.61)	(1.84)	(2.22)	(− 1.70)	(− 1.46)	(− 1.64)
Tech	1.095***	− 5.583***	− 4.488**	0.890***	− 7.149***	− 6.259***	0.994***	0.569***	1.563***
	(7.60)	(− 3.09)	(− 2.48)	(3.59)	(− 3.51)	(− 3.08)	(6.76)	(3.13)	(5.46)
Density	− 0.007**	− 0.056***	− 0.062***	0.047***	0.085***	0.132***	− 0.010***	− 0.006***	− 0.016***
	(− 2.43)	(-3.95)	(− 4.44)	(10.20)	(5.17)	(8.34)	(− 4.29)	(− 2.92)	(− 4.09)

First, the effect of DIF on carbon emissions is tested using Eq. (2). As presented in column (1) of Table 8, the direct and total effects of DIF on carbon emissions are significantly negative at the 5% and 1% levels, respectively, suggesting that DIF can significantly curb carbon emissions. Second, Eq. (3) is utilized to test the effect of DIF on green technological innovation, and column (2) is obtained in Table 8. The findings show that the direct and total effects of DIF on green technological innovation are significantly positive at the 1% level. Meanwhile, the coefficient of the indirect effect is significantly positive at the 1% significance level, indicating a significant spatial spillover. Third, Eq. (4) is applied to analyze the influences of DIF and green technological innovation on carbon emissions. Column (3) of Table 8 reveals that the coefficients of the total effects are significantly negative at the 5% level, suggesting that green technological innovation has a partial mediating effect, accounting for approximately 59.47% of the total effect, as calculated: 1.552 × (− 0.141) / (− 0.368) Table 9.

**Table 9:** Robustness test results.

Variables	(1)	(2)	(3)	(4)
	The explanatory variable lagged by one period	The instrumental variable	Replacement of the spatial weight matrix	Adding control variables
L.DIF	− 0.076***			
DIF		− 0.655***	− 0.085***	− 0.142***
Controls	Yes	Yes	Yes	Yes
Time fixed	Yes	Yes	Yes	Yes
Individual fixed	Yes	Yes	Yes	Yes
R^2^	0.415	0.622	0.515	0.243
Observation	2760	2760	2760	2760

Some helpful conclusions can be drawn from the above analysis of mediating effects. First, DIF can significantly promote green technological innovation and directly reduce carbon emissions. Second, DIF can effectively mitigate carbon emissions in the region by promoting green technological innovation locally. Third, it can be observed that the local DIF can also decrease carbon emissions in neighboring areas by enhancing their green technological innovation, demonstrating significant spatial spillover effects. Consequently, hypothesis 2 is confirmed, suggesting that the growth of DIF in a region not only aids in curbing local carbon emissions but also influences the reduction of carbon emissions in neighboring cities through spatial spillover.

#### Robustness tests

To confirm the robustness of the findings from empirical research, various methods are used for testing, as presented in Tables 9 and 10.

1. The lag period of the explanatory variable^39^. To avoid the problem of reverse causality, DIF is analyzed with a one-period lag. The results indicate that the coefficient of DIF is significantly negative at the 1% level, suggesting the stability of regression results.
2. Estimation of instrumental variables. Referring to Huang et al.^40^, the internet penetration rate (IP) is used in this study as an instrumental variable of DIF to further investigate the potential endogeneity issues. The findings suggest that the coefficient of DIF is significantly negative. Thus, hypothesis 1 holds.
3. Replacement of the spatial weight matrix^41^. The robustness of the spatial model is assessed through the replacement of the spatial weight matrix with an economic geography matrix, thereby reflecting the spatial effect of economic characteristics between regions. The results suggest that the coefficient on DIF is significantly negative, demonstrating the robustness of the dynamic characteristics of the spatial panel model.
4. Adding control variables^42^. To avoid the potential errors from omitted variables, human capital (Edu) and urbanization rate (urban) are added to the regression. The estimation results reveal that there is no significant change in either the estimated value or significance of the DIF coefficient, confirming that the regression results are robust.
5. Re-testing of mediating effects. Considering the potential issue of weak test power in stepwise regression, a Bootstrap^43^ test is conducted. The results in Table 10 indicate that the direct and indirect effects of green technological innovation are significant at the 1% level. The confidence intervals for each pathway exclude 0, substantiating the mediating effect of green technological innovation and reconfirming hypothesis 2.

**Table 10:** Bootstrap mediation test.

Mediating variable	Paths	Effects	Coefficient	Standard error	Confidence intervals	
					Lower limit	Upper limit
lninn	DIF-lninn-CI	Indirect	− 0.041***	0.005	− 0.056	− 0.034
	DIF-lninn-CI	direct	− 0.040**	0.019	− 0.075	− 0.007

#### Spatial heterogeneity analysis

(1) Heterogeneity of urban location

From the perspective of urban location, cities in different regions exhibit varying degrees of economic development. Therefore, to explore how DIF affects carbon emissions across various geographic areas, cities are categorized into eastern, central, and western regions based on geographical location for group analysis. As presented in Table 11, the findings indicate that DIF significantly reduces carbon emissions in eastern cities, but its impact on the central and western regions is insignificant. This disparity may be attributed to the regional variations in the development of DIF in China^44^. Specifically, the eastern regions have outperformed the central and western regions in integrating innovative industries and digital technologies, thus aiding in the reduction of carbon emissions.

**Table 11:** Heterogeneity regression results.

Variables	Location heterogeneity			City size heterogeneity		
	East	Central	West	Small	Medium	Large
DIF	− 0.324***	− 0.003	− 0.293	0.005	− 0.203***	− 0.347***
	(− 5.70)	(− 0.19)	(− 1.12)	(0.27)	(− 7.26)	(− 3.52)
W × DIF	− 1.882***	0.617**	− 0.237	− 0.135*	− 0.628***	− 0.396
	(− 4.08)	(2.41)	(− 1.59)	(− 1.59)	(-2.52)	(− 0.51)
Controls	Yes	Yes	Yes	Yes	Yes	Yes
Time fixed	Yes	Yes	Yes	Yes	Yes	Yes
Individual fixed	Yes	Yes	Yes	Yes	Yes	Yes
N	990	980	790	1740	880	140
R^2^	0.210	0.377	0.171	0.145	0.270	0.535

(2) Heterogeneity in city size

Given the variation in city size, there are significant differences in green technology development and economic resource concentration across cities of different sizes, necessitating heterogeneity tests categorized by city size. The results show that DIF significantly reduces carbon emissions in large and medium-sized cities. However, the effect of reducing carbon emissions in small cities is insignificant. This can be attributed to the availability of more comprehensive inclusive financial services and advanced digital infrastructure in large and medium-sized cities, which enhance green technological innovation and foster digital industry growth, thereby optimizing the industrial structure and reducing carbon emissions. Conversely, the insufficiency of digital technology and inclusive financial development in small cities does not distinctly manifest a similar inhibitory effect on urban carbon emissions.

#### Further discussion: spatial threshold effects

To further investigate the nonlinear impact of DIF on carbon emissions, the threshold effect model based on the spatial lag term is constructed^45^, set as follows:

6 $$ \begin{aligned} CI_{it} &= \omega_{0} + \omega_{1} w_{ij} DIF_{it} \cdot I(T_{it} < \delta_{1} ) + \omega_{2} w_{ij} DIF_{it} \cdot I(\delta_{1} \le T_{it} < \delta_{2} ) \\ & \quad + \omega_{3} w_{ij} DIF_{it} \cdot I(T_{it} \ge \delta_{2} ) + \gamma X_{it} + \varepsilon_{it} \end{aligned}$$

In Eq. (6), $T$ represents the threshold variable, $\delta$ denotes the threshold value, and $I$ indicates the indicator function, which takes the value 1 when a specific condition is met and 0 otherwise.

From the previous research analysis, it is evident that the impact of DIF on carbon emissions is influenced by the upgrading of industrial structures and green technological innovation. Therefore, industrial structure upgrading and green technological innovation are designated as threshold variables, with their threshold values estimated through Bootstrap resampling, as detailed in Table 12. The results show a single-threshold effect in industrial structure upgrading, with a threshold value of 1.022, and a double-threshold effect in green technological innovation, with threshold values of 1.129 and 1.497, respectively.

**Table 12:** Threshold test results.

Threshold variables	Number of thresholds	F-statistic	P-value	10%	5%	1%	Threshold value
Stru	Single	66.17***	0.010	33.890	36.196	43.973	1.022
lninn	Single	57.47***	0.010	36.979	41.249	53.471	1.129
	Double	54.86***	0.010	28.780	39.495	51.591	1.497

**Figure 3:**
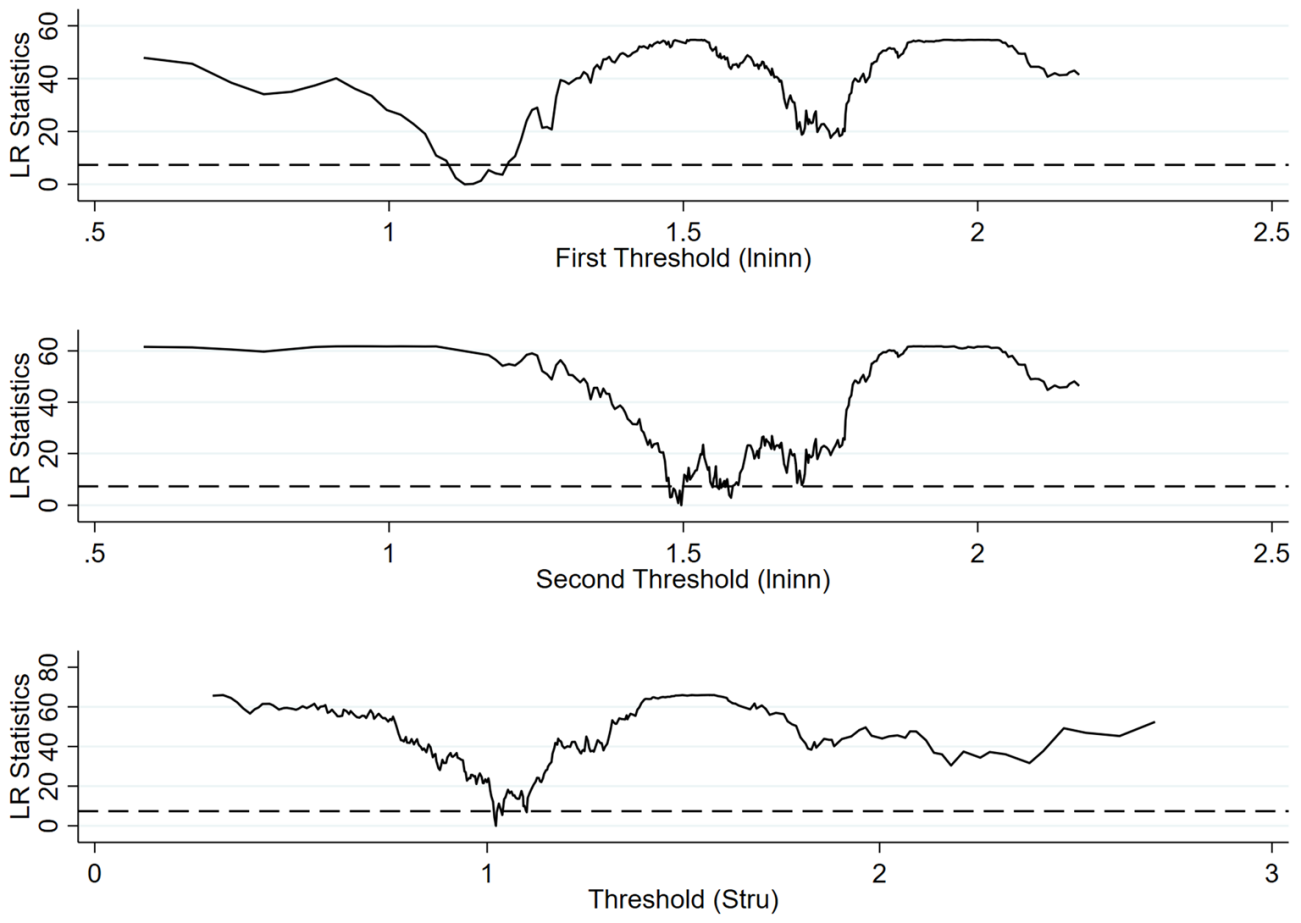
Threshold estimates and confidence intervals.

Table 13 presents the threshold regression results, and Fig. 3 depicts the relationship between the threshold estimates and the LR statistics. The results indicate that the inhibitory effect of DIF on carbon emissions gradually strengthens as the upgrading of industrial structures surpasses the threshold value. Specifically, when the industrial structure upgrading is below the threshold value, DIF exhibits significance at the 5% level, with a coefficient of − 0.082, signifying that DIF reduces carbon emissions. Moreover, when industrial structure upgrading surpasses the threshold value, the coefficient of DIF, significant at the 1% level, is − 0.124, suggesting a significant reduction of carbon emissions by DIF. This may be attributed to the increasing investment initiatives by the government and financial sector in upgrading industrial structures, facilitating the green transformation of energy-intensive industries, enhancing energy efficiency, and decreasing carbon emissions.

**Table 13:** Threshold regression results.

Threshold variables	Stru	lninn
W.DIF × I(Stru < 1.022)	− 0.082**	
	(− 2.00)	
W.DIF × I(Stru ≥ 1.022)	− 0.124***	
	(− 3.09)	
W.DIF × I(lninn < 1.129)		− 0.265***
		(− 6.26)
W.DIF × I(1.129 ≤ lninn < 1.497)		− 0.202***
		(− 4.97)
W.DIF × I(lninn ≥ 1.497)		− 0.159***
		(− 3.97)
Controls	Yes	Yes
Time fixed effect	Yes	Yes
Individual fixed effect	Yes	Yes
R^2^	0.610	0.628
Observation	2760	2760

The impact of DIF on carbon emission reduction exhibits a marginally diminishing trend with the improvement of green technological innovation. First, when green technological innovation is below the threshold value, DIF significantly impacts at the 1% level, with a coefficient of − 0.265. This suggests that DIF can significantly promote the reduction of carbon emissions. Second, when the threshold value ranges between 1.129 and 1.497, the impact coefficient is − 0.202. The inhibitory effect is weaker compared to the first stage. Third, when green technological innovation surpasses the threshold value, DIF is significant at the 1% level, with an estimated coefficient of − 0.159. This inhibitory effect is further reduced compared to the second stage. This may be attributed to the different development stages of green technological innovation. Specifically, at the early stage of green technological innovation, traditional industrial development exhibits low productivity and energy utilization rates. By promoting R&D in green and clean production technologies, DIF significantly improves the efficiency of resource allocation and energy utilization in social production, thereby leading to substantial reductions in carbon emissions. However, as development progresses to the middle and later stages, the green technology market often saturates and matures, leaving limited room for innovation. At this time, further reductions in carbon emissions may require greater investment in the development of more advanced and sophisticated technologies or the implementation of mandatory external measures, such as government regulations. This may lead to a gradually decreasing potential for carbon emission reductions through the advancement of green technological innovation. Therefore, hypothesis 3 is supported.

## Conclusions and policy recommendations

Utilizing panel data from 276 Chinese cities from 2011 to 2020, this study empirically investigates the impact of DIF on carbon emissions and the intrinsic mechanism of green technological innovation from a spatial perspective by employing various methods. The following research findings are obtained:

(1) DIF has a negative spatial spillover effect on carbon emissions, promoting the reduction of local carbon emissions and significantly reducing emissions in neighboring regions. (2) Sub-dimensional tests demonstrate that the breadth and depth of DIF significantly reduce carbon emissions. (3) Regarding the transmission mechanism, green technological innovation has a significant indirect contribution to the inhibitory effect of DIF on carbon emissions from a spatial perspective. (4) Heterogeneity analysis reveals more significant carbon reduction effects of DIF in the eastern regions and large and medium-sized cities. (5) Nonlinear tests indicate that the inhibitory effect of DIF on carbon emissions is influenced by green technological innovation and industrial structure upgrading, showing gradual weakening and strengthening, respectively.

Based on the empirical evidence from this study, the following policy recommendations are proposed. (1) The expansion and deepening of DIF across regions should be strengthened. Given the effectiveness of DIF in reducing urban carbon emissions and its evident spatial spillover effect, governments are urged to facilitate its widespread adoption and application across cities, thus enhancing both its breadth and depth. Furthermore, by leveraging the regional allocation function of financial services to serve underserved populations and integrating digital technologies, significant reductions in carbon emissions can potentially be achieved within the region and through spillover effects to adjacent areas. (2) The development of green technological innovations should be emphasized. Governments should encourage the integration of green financing in DIF platforms and support the expansion of green technological innovation projects for startups. By implementing incentive measures to cultivate regional innovation clusters focused on green technology, these clusters can exemplify best practices for neighboring regions, thereby enhancing the research and application of green technology to reduce carbon emissions. (3) Implement dynamic and differentiated strategies for the growth of DIF. Tailored economic policies should be formulated to maintain the advantages of digital finance in medium and large cities and the eastern region. Innovations in financial services should be promoted. For local governments in the central and western regions and small cities, the construction of digital infrastructure should be enhanced, facilitating resonance and synergy between artificial intelligence and inclusive financial services. This will guide the allocation of factor resources towards low-carbon and environmentally friendly industries and jointly advance urban carbon emission reduction efforts. (4) Targeted development strategies for green technological innovation and industrial structure upgrading. Policymakers should not only maximize the environmental benefits of DIF but also develop policies aligned with the stages of green technological innovation and industrial structural upgrading. For example, DIF can be directed to strongly support the initial R&D in industries dedicated to green technological innovations. As these industries achieve maturity in applying green technologies, incentives can be gradually diminished, thereby optimally utilizing the carbon emission reduction potential of DIF. Furthermore, DIF can gradually reduce carbon emissions by encouraging industrial structure upgrading, eliminating highly polluting, outdated production capacities, and supporting the advancement of low-end industries.

However, the limitations of this study may provide valuable insights for future research. First, this study primarily examines the intrinsic mechanism of green technological innovation. Notably, the impact of DIF on carbon emissions involves multiple pathways. A detailed discussion is limited by the length of this study. Future research could investigate the mechanisms involving factors like consumption patterns and income levels. Second, regarding the metric measurement, this study focuses on carbon emission indicators from the perspective of the formation source of carbon dioxide. In the future, a more comprehensive exploration can be conducted based on factors such as energy combustion consumption and the input and output of industries.

## Data Availability

Publicly available datasets were analyzed in this study. This data can be found here: (1) https://idf.pku.edu.cn/. (2) http://www.cnstats.org/tjnj. (3) https://www.cnrds.com/.
